# Sex differences in locus coeruleus physiology

**DOI:** 10.1002/alz.085461

**Published:** 2025-01-03

**Authors:** Rosaria Josephine Rae, Lori L McMahon

**Affiliations:** ^1^ Medical University of South Carolina, Charleston, SC USA; ^2^ The Medical University of South Carolina, Charleston, SC USA

## Abstract

**Background:**

Alzheimer’s disease disproportionately affects women in the U.S., with two‐thirds of individuals diagnosed being female. This gender disparity underscores the crucial role of sex as a significant risk factor for disease development. Recognizing the importance of this, it becomes imperative to investigate how Alzheimer’s disease presents differently between males and females, especially in the early stages when intervention is most potent. The locus coeruleus (LC) is the initial brain region affected by hyper phosphorylated tau in Alzheimer’s disease, preceding the accumulation of other pathological indicators. A deeper understanding of sex differences in this crucial region can provide insights into how Alzheimer’s disease related dysfunction manifests uniquely between the sexes

**Method:**

In vivo electrophysiology under anesthesia was used to measure single unit activity of LC firing patterns in wild‐type Fischer male and female rats. Females have been estrous cycle tracked and stratification between estrus phases may uncover further differences within females. Recordings measured tonic firing and phasic firing in response to footshock. Spike sorting and signal processing differentiated LC principal neurons and interneurons, unique units, physiological features, and bursting patterns that were compared between groups.

**Result:**

This data shows no significant differences in LC principal cell tonic firing between males and females. However during burst firing, females have an increased interspike interval, indicating decreased firing supporting sexually distinct physiology of the LC. Additionally, females have distinct waveform patterns from males including area under the curve and peak/valley energies, suggesting possible differences in intrinsic properties.

**Conclusion:**

Sex influences LC phasic physiology which may contribute to AD disproportionately impacting women.

